# Unraveling the Complexity of Apert Syndrome: Genetics, Clinical Insights, and Future Frontiers

**DOI:** 10.7759/cureus.47281

**Published:** 2023-10-18

**Authors:** Kajol Kumari, Inam Saleh, Sanzida Taslim, Sana Ahmad, Iqbal Hussain, Zainab Munir, Tamleel Javed, Muhammad Furqan Ismat Virk, Saleha Javed, Pakeezah Bisharat, Ubaid Ur Rehman

**Affiliations:** 1 Dentistry, Jinnah Sindh Medical University, Karachi, PAK; 2 Paediatrics, University of Kentucky College of Medicine, Lexington, USA; 3 Psychiatry, Ross University School of Medicine, Bridgetown, BRB; 4 Psychiatry, TIME Organization, Inc., Baltimore, USA; 5 Internal Medicine, Khyber Medical University, Peshawar, PAK; 6 Internal Medicine, Lady Reading Hospital, Peshawar, PAK; 7 Emergency Department, Imran Idrees Teaching Hospital, Sialkot, PAK; 8 Paediatrics, ABWA Hospital and Research Centre, Faisalabad, PAK; 9 Emergency Department, Sheikh Zayed Hospital, Rahim Yar Khan, PAK; 10 Internal Medicine, Khyber Medical University, Peshawar , PAK; 11 Internal Medicine, Mayo Hospital, Lahore, PAK

**Keywords:** future research, quality of life, psychosocial considerations, medical management, diagnosis, genetics, clinical manifestations, fgfr2 gene, craniosynostosis, apert syndrome

## Abstract

Apert syndrome (AS), also known as type I acrocephalosyndactyly, is a rare congenital condition characterized by craniosynostosis resulting from missense mutations in the fibroblast growth factor receptor 2 (*FGFR2*) gene. This comprehensive review delves into AS, covering its clinical manifestations, genetics, diagnosis, medical management, psychosocial considerations, and future research directions. AS presents with distinct features, including a brachycephalic skull, midface hypoplasia, and limb anomalies such as syndactyly. It follows an autosomal dominant inheritance pattern with mutations in the *FGFR2* gene. Prenatal diagnosis is possible through advanced imaging techniques and molecular testing. The multidisciplinary approach to AS management involves surgical interventions, orthodontics, and psychological support. Although no curative treatment exists, early interventions can significantly improve function and aesthetics. The quality of life for AS patients is influenced by psychosocial factors, necessitating comprehensive support for both patients and their families. Future research directions include gene therapy, understanding cellular responses to *FGFR2* mutations, and addressing genetic heterogeneity. Collaborative efforts are vital to advancing knowledge about AS and its genetic underpinnings. Overall, this review serves as a valuable resource for healthcare professionals, educators, and researchers, contributing to a deeper understanding of AS and facilitating advancements in diagnosis and treatment.

## Introduction and background

Apert syndrome (AS), also known as type I acrocephalosyndactyly, is a rare congenital condition of craniosynostosis caused by missense mutations in the fibroblast growth factor receptor 2 (*FGFR2*) gene. A brachycephalic skull, midface hypoplasia, and limb anomalies like syndactyly of the hands and feet define this disorder. In addition to these main traits, the syndrome manifests itself in many ways in the oral/maxillofacial region, bones, brain, skin, and internal organs [1]. Initially described by Wheaton in 1894, the French pediatrician Eugene Apert's 1906 paper described a group of nine cases demonstrating the distinct triad of maxillary hypoplasia, syndactyly, and craniosynostosis, which led to the namesake association [2].

The syndrome shows an autosomal dominant pattern, which can be tied to advanced paternal age, maternal infections, maternal drug exposure, and inflammatory cerebral processes. The *FGFR2* gene-specific missense mutations on chromosome 10q25-10q26 make up the majority of cases (more than 98%) [3]. Mouse models have greatly facilitated the research of this rare disorder's complexities, aiding in our understanding of it [4]. Similar skeletal dysplasias or craniosynostoses, such as Crouzon, Pfeiffer, Carpenter, Jackson-Weiss, Beare-Stevenson, Saethre-Chotzen, cloverleaf skull, Vogt cephalodactyly, and FGFR3 coronal synostosis syndromes, can be confused for AS. Therefore, it is advised to confirm the diagnosis by molecular genetic mapping of certain *FGFR* gene mutations or by prenatal sonographic identification of structural defects [1].

The main objective of this review is to thoroughly discuss the complexities of AS. It aims to shed light on the multidisciplinary nature of AS, covering the current understanding of genetic alterations, pathophysiology, clinical manifestations, psychosocial considerations, potential therapeutic approaches, craniofacial surgery, orthopedics, and quality of life, through the analysis of various clinical cases and related research findings. The ultimate goal is to serve as a useful resource for educators, researchers, and healthcare practitioners alike, to encourage a deeper understanding of AS and facilitate improvements in its diagnosis and treatment modalities.

## Review

Epidemiology and genetics

AS accounts for 4.5% of all occurrences of craniosynostosis, with an incidence rate of 6-15 cases per one million live births [5]. Its prevalence spans without a gender-based predilection. The condition follows an autosomal dominant pattern of inheritance and is linked to several things, including maternal illnesses, maternal drug exposure, advanced paternal age, and cranial inflammatory processes [1].

The *FGFR2* gene, which is located at chromosome 10q26, is the genetic basis for AS [2]. With three immunoglobulin (Ig)-like domains in its extracellular domain, a transmembrane segment, and an intracellular tyrosine kinase domain, *FGFR2* is a member of the family of transmembrane tyrosine kinase receptors. Through downstream processes, this receptor integrates the transmission of signals from extracellular to intracellular areas, consequently regulating a variety of cellular processes. The expression of FGFR2 is found in a variety of tissues, including the brain, lungs, skin, limb mesenchyme, proliferating osteoprogenitors, and early cartilage condensations in the embryo. The two isoforms produced by this gene are IIIb, which is expressed in epithelial tissue, and IIIc, which is expressed in neural tissue and mesenchyme. These isoforms control normal organ development through their correspondence to particular FGF ligands [6]. Studies show that 98% of the incidents result from one of two heterozygous mutations in the *FGFR2* gene, which encode the amino acid substitutions Ser252Trp (the S252W mutation) or Pro253Arg (the P253R mutation) [4]. The *FGFR2* mutations are solely paternal. AS carriers have a 50% chance of passing the condition on to their children, with a 1% chance that a second kid would also be affected. The *FGFR2* gene regulates cell proliferation and survival, which affects the production of essential fibrous materials during the development of craniofacial tissue, bone sutures, cartilage, and tooth regeneration, and is responsible for the manifestations of AS [1].

About two-thirds of patients with unrelated AS have the S252W mutation, whereas the remaining one-third have the P253R mutation [6]. While P253R is linked to severe syndactyly, S252W, the more common mutation, is linked to severe craniofacial deformities. These mutations largely affect the area of FGFR2 that connects immunoglobulin-like domains II and III, resulting in modified specificity and enhanced ligand binding affinity. Due to this disruption, improper cell migration, proliferation, and differentiation occur, which leads to early osteogenesis and the distinctive skeletal deformities that accompany AS [2].

First discovered in 1995 were the alterations ser252trp of 755C>G (S252W) and pro253arg of 758C>G (P253R). An additional investigation revealed a novel ser252phe mutation in the *FGFR2* gene, comprising a shift from CG-to-TT in the cDNA. Moreover, a 2002 genomic evaluation of FGFR2 revealed an M186T (c.557C>T) mutation in AS. Following this, additional uncommon variants were found, including 940-2A_G, 940-3_-4insAlu, and 1041_1042insAlu. Just a while back, a Korean patient with AS was found to have the unique E731K (c.2191G>A) mutation within exon 18 of the *FGFR2* gene [6].

Clinical presentation

The patient often exhibits symmetrical syndactyly of the second, third, and fourth fingers and toes on both the hands and feet, with partial or total union of the skin and bones of the fingers and toes with a common nail [7,8]. Acrocephaly, brachycephaly, flat occiput, prominent forehead, hypoplastic midface, and a vertically exaggerated craniofacial complex are the outcomes of early synostosis of the cranial base in combination with coronal synostosis and sagittal and metopic suture agenesis [9]. Palpebral fissures that point downward, hypertelorism, a shallow orbit, proptosis, and exophthalmos are all observed in the eyes [10]. Optic atrophy due to papilledema may also occur [11]. There have also been reports of strabismus (60%), anisometropia (19%), amblyopia (14%), and ametropia (34%) [12]. The nasal bridge of the nose is unquestionably flat. The maxilla is also retro-positioned and hypoplastic. Due to inadequate aeration in the maxillary antra, the palate is narrow and high-arched [10]. The central trough of the palate is highly noticeable and challenging to clean because of bulbous lateral palatal swellings. It is fairly common to have a pseudo-cleft palate and an anteriorly pointed palatal plane [13]. The maxillary arch is V-shaped and slopes down posteriorly, leading to an anterior open bite. Delay in eruption, impactions, thick gingiva, severe crowding of developing teeth within the alveolus, and sometimes surplus or congenitally absent teeth are observed [14].

In 40% of instances, patients also present with disorders of the lower respiratory tract, such as choanal stenosis, tracheal cartilage anomalies, and some degree of air blockage [15]. Due to decreased airway patency, people become mouth breathers out of necessity, and there is a consequent anterior open bite [16].

Up to 71% of patients with AS experience cervical spine fusion, which most frequently affects the fifth and sixth vertebrae [17]. Of the patients, 10% come up with cardiovascular anomalies, while 9.6% show genitourinary defects [18]. Cardiovascular manifestations can include anomalies like atrial septal defect (ASD), ventricular septal defect (VSD), patent ductus artery (PDA), and pulmonary stenosis [11].

Intelligence ranges from normal to subnormal [11]. Before the pathognomonic skeletal alterations are evident, some fetuses with AS may exhibit central nervous system anomalies, including mild ventriculomegaly or corpus callosum agenesis. Also documented were cases with even more unusual appearances, like an omphalocele or diaphragmatic hernia [19]. Additionally, acne, hypopigmentation, hyperhidrosis, and hyperkeratosis of the plantar surfaces have been recognized in the literature as cutaneous signs of AS [20]. 

Diagnosis and differential diagnosis

While it is possible to some extent to generalize the usual AS symptoms, each affected child has a distinctive presentation that must be considered when developing preventive and therapeutic efforts [21]. A child with AS is often diagnosed through clinical detection and additional confirmation calls for molecular diagnostic tests. Since patients with AS have compromised cranial bones and the resultant clinical overlap with those who have Pfeiffer syndrome and Crouzan syndrome, research using whole-exome sequencing can be very useful in providing significant information about these rare genetic disorders [4,22].

A prenatal diagnosis of AS can be made with the aid of three-dimensional sonography, MRI, and fetoscopy at mid-trimester [23]. Fetoscopy was first used to diagnose AS in utero. Prenatal ultrasound has recently been used for third-trimester diagnoses when it is more probable to find anomalies in the shape of the skull associated with craniosynostosis [24]. A definitive antenatal diagnosis has been made possible by the identification of *FGFR* gene mutations. However, in the majority of instances, a complete clinical evaluation and specialized testing, including the molecular genetic test, are used to confirm the diagnosis at birth or during the first few months of infancy [23]. In the early prenatal diagnosis of this condition, ultrasound and MRI appear to play complementary roles [25,26].

Before the third trimester, craniosynostosis disorders are difficult to diagnose. Many cases are either not discovered until delivery or are only discovered in late pregnancy when craniofacial malformations are more prominent. Consequently, even though this condition is characterized by a particular combination of abnormalities, the diversity of its clinical presentation and the challenges associated with its early detection make the diagnosis challenging [27].

Until recently, only familial cases could be diagnosed with confidence based on early-pregnancy ultrasound evidence. Before prenatal molecular testing, the majority of the more frequent sporadic instances went undiagnosed until the third trimester, when the syndrome's full picture generally emerged. Clinical confirmation at birth was the ultimate diagnosis. However, the fusion of two crucial elements leads to an accurate diagnosis of AS. First, to discover the subtle sonographic signals that point to a monogenic condition, an understanding of genetic disorders and dysmorphology is essential. Second, the availability of molecular testing has changed how we handle AS by enabling accurate genetic counseling and a firm diagnosis [24].

Other cranial synostosis syndromes such as Crouzon, Pfeiffer, Carpenter, and Saethre-Chotzen are included in the differential diagnosis of AS. Except for Carpenter syndrome, which is linked to mutations in RAB23 (Ras-associated protein), all craniosynostosis syndromes result from *FGFR2* gene mutations. All of them share several clinically comparable symptoms as well. The facial deformity in Crouzon syndrome is typically milder than the one in AS, and deformities in the hands and feet are infrequent. In contrast to AS, Pfeiffer syndrome commonly involves skeletal (such as radiohumeral synostosis of the elbow), central nervous system (such as hydrocephaly), and gastrointestinal abnormalities (such as imperforated anus). Similar to AS, Pfeiffer and Saethre-Chotzen syndromes can also cause syndactyly. However, unlike AS, it is usually partial. Syndactyly is indeed a significant trait of AS and is typically complicated, with the index, middle, ring, and fifth fingers sharing a fused bone (mitten hands), which is why it can be highly helpful in distinguishing it from other craniosynostosis disorders [27,28]. 

Medical management

Patients with AS typically require lifetime care, although there are currently no effective treatment modalities other than surgery [6]. Following an appropriate diagnosis, treatment should begin immediately after birth. A multidisciplinary (respiratory, cerebral, dental, ocular, and orthopedic) approach is necessary since the syndrome impacts multiple systems. There is a need for both surgical and supportive care. To avoid issues like high intracranial pressure and premature suture closure, an early craniotomy is necessary. Surgical finger separation and cosmetic face reconstruction can be done to improve both function and aesthetics. To effectively rehabilitate the child in society, early hearing optimization with potential hearing aids, airway management, psychological counseling, speech correction, and genetic counseling are necessary [29]. Typically, a craniotomy is performed at the age of six months to treat craniosynostosis and brain compression. Corrective surgery for hand or foot digit syndactyly is performed between the ages of one and four years; midface and palate corrections are performed at the age of four to six years and orthognathic surgery or orthodontics are performed during the permanent dentition stage or after growth completion [30]. Orthodontic therapy and orthognathic surgery can be used to treat severe skeletal class III open bite malocclusion in patients. Later, conventional surgical advancement of the midface (Le Fort III osteotomy) or progressive advancement with osteogenic distraction (Ilizarov technique) can be performed [29].

By educating parents on alternative AS treatment options and proper nutrition or hygienic lifestyles, pediatric dentists can start their patients' professional oral care from the beginning, even before the child is born. It is highly beneficial to schedule routine checkups, hygiene prophylaxis, fluoride treatments, pit and fissure sealants, and preventative or interceptive orthodontic techniques following the eruption of the first teeth [31]. The individuals' hand abnormalities, malocclusion, and pseudo-cleft in the palate make it difficult for them to maintain oral hygiene. The work might be made simpler by fluoride mouthwashes, new-generation electric toothbrushes, or custom-designed toothbrushes [2].

If possible, physical deformity management should begin in childhood; nonetheless, adult patients may have orthodontic and surgical therapy in the future [32]. Surgical correction of the craniofacial region and fingers and toes may result in considerable functional and aesthetic improvement when diagnosed in the patient as early as three months of age. The aberrant neurological development exhibited in mental deficiency may be one of several consequences that occur if the AS is detected later in life or if treatment is postponed. Facial abnormalities, pseudo-prognathic mandibles, or class III malocclusion tend to worsen with age, and the maxilla slants down posteriorly; also, finger function deteriorates with slowed skeletal growth and other deformities. Luckily, most children with AS don't need any special dietary advice or activity or sports limitations [7,33]. 

Typically, structural defects linked to AS can be detected during pregnancy using sonography. The doctor should advise the parents that the prognosis is poor (increased chance of intellectual disability and several postnatal procedures), so that they may choose to terminate the pregnancy before the fetal viability stage [34].

Psychosocial and quality of life considerations

As a consequence of AS, patients suffer from lifelong dependence. This is a regrettable social outcome that begins with developmental difficulties experienced in childhood and gets worse with adult social maladjustment. In comparison to their normal counterparts, they are less educated, less frequently married, have fewer acquaintances, and live with their parents in the majority of cases [35]. Despite having a full-scale intelligence quotient comparable to the normative population, children with syndromic craniosynostosis are more likely to experience intellectual disability and social issues [36]. This highlights the need for early and thorough rehabilitation to improve their quality of life. Sadly, there aren't enough systematic and coordinated medical and psychosocial therapies in locations with low resources to handle cases or help families deal with their difficulties [37]. 

The impact of hereditary illnesses on the family spans the patient's lifetime and has several dimensions. Parents coping mechanisms are put under a great deal of strain when a child is born with craniofacial defects [38]. Families of newborns with AS are frequently upset by the care requirements imposed by this craniofacial abnormality and are let down by an utterly unanticipated situation [39]. The acquisition of cognitive skills to manage the novel scenario involving intimate contact between parent-child and child-peers is facilitated by psychological support for patients and parents [40]. Referring patients to support groups so they can obtain individualized care and family assistance is advantageous; in industrialized nations, support groups for parents of children with syndromes like AS will give counseling opportunities to share and discuss parental concerns. The severity of AS presentation, as well as the effectiveness and timing of treatment, all influence a patient's lifelong prognosis. Prenatal diagnosis may be aided by genetic consultation and screening; if patients are continuously monitored for developmental delays, early intervention therapy may be used as needed. When the kid reaches school age, a psychological assessment can be planned to identify difficulties with language, social-emotional development, and/or cognitive or academic skills and implement an intervention plan [37]. 

Since quality-of-life measures are currently the accepted gold standard for patient-reported measurement outcomes, they may enable a comprehensive understanding of the effect of treatment on patients' lives [41]. There have only been a few studies done so far to evaluate the quality of life for people with AS. Patients with AS who are born in impoverished areas can have difficulty finding the right care in a specialty hospital. In more affluent and developed areas, families typically cannot afford transportation and maintenance. Local administrative districts in those areas refuse to provide financial support for treatment outside of their territory because they believe their region already provides adequate healthcare. One of the most difficult hurdles to cross in treatment is this indication of ignorance regarding the complexities of this condition. As a result, it is not unusual for these individuals to show up late, presenting with an even more complex situation that may include intellectual disability, blindness, and hand sequelae with significant bony distortion. Depending on the patient's age and the degree of the deformity at the initial visit, there may not be much that can be done, despite the family's desperate wishes. The patients surprisingly display an adequate level of functioning despite the obvious craniofacial deformity and physical restrictions of the upper and lower limbs. Contrary to all the potential negative effects and restrictions that AS may impose on daily activities, children who are born with the condition appear to benefit from psychological and familial support. The family and the craniofacial team must exert tremendous effort to provide them with surgical care and comprehensive rehabilitation [42].

Future directions and research

Understanding how mutations affect signaling pathways and interactions with effector molecules that result in improper craniofacial development and other growth disorders has been made possible by experiments utilizing mutant mouse models from outbred and inbred backgrounds [43]. The research of mouse models is crucial to understanding the symptom variation and the similarities between AS transgenic mouse models and AS patients at the molecular, histological, and morphological levels [44].

The understanding of the pathophysiology of AS has been significantly advanced by the application of cutting-edge techniques such as conditional targeting and knock-out technologies, differential gene expression, tissue culture investigations, three-dimensional detection approaches, and other advanced imaging techniques. The majority of these procedures cannot be replicated in humans or cell lines, but in certain suspected cases, molecular tools like sequencing, microarray, exome sequencing, and deep next-generation sequencing can identify fascinating genetic modifications responsible for individual phenotypic differences [4].

Gene therapies are significantly promising for curing diseases that were previously incurable. To revolutionize therapies for a variety of ailments, manufacturers are investing more money in this area. This method may reduce the need for surgery by preventing, treating, or curing underlying illnesses. Importantly, the lack of published molecular genetic information on craniosynostosis in the literature has emerged as one of the major obstacles to the advancement of a genomic strategy for precision medicine. It is advised that more research on molecular genetics in the area of syndromic craniosynostosis is necessary before gene therapy can be a part of an interventional strategy in AS. Clinical trials are also required to evaluate gene therapy in AS patients [45].

A long-term effect is something that various gene treatment strategies try to achieve in terms of persistence and integration. Wherever this kind of durability is necessary, the therapeutic material must continue to work for the specified time. According to reports, there are two ways to accomplish this: either use numerous rounds of gene therapy to keep the therapeutic genes active for a longer period, or combine the therapeutic genes [46]. Targeting the FGF/FGFR pathway with biological therapies can result in an excellent prognosis. In the cultured calvaria of *FGFR2*+/P253R mice, PD98059, an MEK1 inhibitor, has been observed to lessen coronal suture fusion [47]. Additionally, it has been found that a soluble version of *FGFR2* with the Ser252Trp mutation can somewhat alleviate the Apert mouse model phenotype by preventing the early closure of the coronal suture in cultured calvaria and transgenic mice [48,49]. According to a study, inhibiting MEK1/2 with U0126 helps *FGFR2* ± S252W mice with their craniosynostosis phenotypes [50]. Adeno-associated virus-mediated RNAi (S2) has been observed to suppress the protein levels of FGFR2, phosphorylate ERK1/2, and P38, and decrease the levels of mutant *FGFR2*, Runx2, collagen 1, osteocalcin, and osteopontin, which led to a reduction in suture fusion [51]. Tamoxifen has been seen to elevate p-ERK1/2 MAPK and the expression of Runx2, Opn, ALP, Col1A1, osteocalcin, and the Rankl/Opg ratio, leading to the appropriate closure of coronal sutures [52]. Juglone (5-hydroxy-1,4 naphthoquinone; 420120, Calbiochem) is a pharmacological inhibitor that has been shown to decrease RUNX2, block downstream Dusp6 and Spry2, and limit the expression of Cyclin D1, Cdk2, Cdk4, and PCNA, leading to the normal closure of coronal sutures [53]. Additionally, it caused the expression of type II collagen alpha 1 and type X, which in turn prevented the union of sutures in mice [54]. Despite the substantial knowledge of cell behavior alterations caused by the *FGFR2* mutation, different cell types and differentiation stages respond differently to the activated *FGFR2* mutation in AS, which requires further exploration in the future [6].

## Conclusions

This review meticulously evaluated AS, a rare congenital condition of craniosynostosis caused by mutations in the *FGFR2* gene. A distinctive trio of clinical characteristics, including a brachycephalic skull, midface hypoplasia, and limb anomalies like hands-and-feet syndactyly, characterize AS. The importance of early diagnosis using clinical assessment, molecular genetic testing, and prenatal imaging techniques has been emphasized in the article. For best treatment results, AS patients require multidisciplinary care that includes orthopedics, craniofacial surgery, orthodontics, and other disciplines. Surgical procedures are crucial to treating craniofacial and limb malformations. The disorder's psychosocial ramifications are highlighted by the difficulties that AS patients and their families endure, which call for psychological and family assistance. Access to comprehensive care is impacted by resource constraints in some situations, stressing the need for appropriate delivery of healthcare. Future research directions have also been outlined in the article, including gene therapies, comprehending cellular reactions to *FGFR2* mutations, and dealing with genetic heterogeneity. Collaborative efforts are essential for furthering our understanding of AS and its genetic basis.
